# Spigelian Hernia: A Case Report

**DOI:** 10.31729/jnma.8440

**Published:** 2024-02-29

**Authors:** Prinsa Shrestha, Gaurab KC, Bidur Acharya, Shiv Raj Shah, Sujan Regmee

**Affiliations:** 1 Department of General Surgery, Kathmandu Medical College and Teaching Hospital, Sinamangal, Kathmandu, Nepal

**Keywords:** *case reports*, *rectus abdominis*, *ventral hernia*

## Abstract

A Spigelian hernia is a hernia through the Spigelian fascia which are difficult to diagnose as they do not present with a subcutaneous swelling and can be dangerous as there is a high risk of incarceration. We report a case of a 51-year-old female who presented to our surgical unit with epigastric pain for 5 days. She was diagnosed with Spigelian hernia with esophagitis and antral gastritis with the help of a computed tomography scan and upper gastrointestinal endoscopy. The diagnosis was confirmed on diagnostic laparoscopy and transabdominal preperitoneal repair of the defect was performed using prolene mesh. Her post-operative period was uneventful. Spigelian hernias are rare and patients can present with atypical symptoms as in this case. Thus, imaging plays a vital role in diagnosis. Management is surgical and has good outcomes.

## INTRODUCTION

Spigelian hernia arises through a defect in Spigelian fascia; which is the aponeurotic layer between the rectus abdominis muscle medially, and the semilunar line laterally.^1^ It is often difficult to diagnose this condition preoperatively; however, the use of ultrasonography (USG) and computed tomography (CT) scans has aided in the diagnosis of this rare hernia and planned surgical management.^2-4^ We report a case of a 50-year female, who presented to our surgical outpatient department with an atypical presentation of epigastric pain for 5 days with no notable swelling in the abdomen. The diagnosis of a Spigelian hernia was made with the help of a contrast-enhanced CT scan and confirmed on exploration.

## CASE REPORT

A 51-year-old lady presented to our surgical outpatient department with the chief complaint of abdominal pain in the epigastric region for 5 days, which was gradual on onset, burning in nature, exacerbated by meals, without radiation, and relieving factors. There was no history of nausea, vomiting, abdominal distension, constipation, or any visible lumps in the abdomen. However, she had a history of retching, belching, and water brass. There was no history of other systemic symptoms. The patient is an ex-smoker and consumes alcohol occasionally. There was no history of predisposing factors of hernia like chronic cough, constipation, urinary retention, etc. However, there was a history of total abdominal hysterectomy 6 years back.

On examination, she was hemodynamically stable with normal general physical examination. On abdominal examination, there was no visible swelling; however, on deep palpation, a non-tender swelling measuring approximately 3 x 3 cm in size was palpated in the right infra-umbilical region with the presence of cough impulse. The rest of the systemic examinations were unremarkable. Various investigations including baseline blood investigations, ultrasound of the abdomen and pelvis, upper gastrointestinal (UGI) endoscopy, and contrast-enhanced computed tomography (CECT) of the abdomen and pelvis were done. Hemograms, renal function tests, urinalysis, and routine stool tests were within normal limits. Serum lipase and amylase levels were also within normal limits.

**Figure 1:**
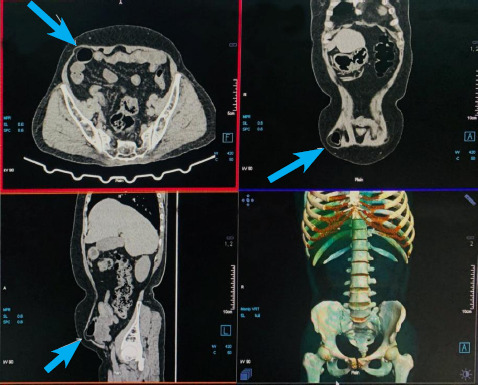
CT of the patient (arrow showing Spigelian hernia).

UGI endoscopy revealed esophagitis of Los Angeles (LA) grade A with antral gastritis. Ultrasonography of the abdomen and pelvis revealed normal findings while CECT revealed a defect measuring 26.3 × 24.8 mm at Spigelian fascia with herniation of omental fat and small bowel loop suggesting Spigelian hernia (Figure 1).

**Figure 2:**
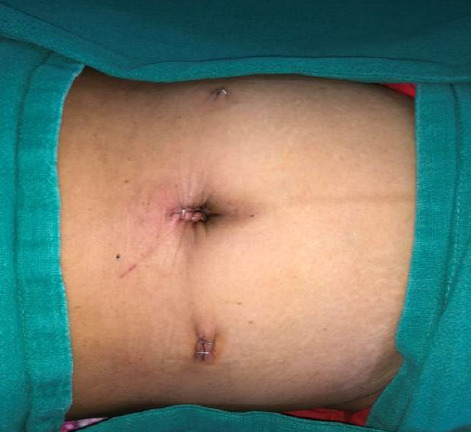
Showing laparoscopic ports position.

She was admitted and planned for laparoscopic repair under general anaesthesia. After pre-operative evaluation, she was managed with transabdominal preperitoneal (TAPP) repair. Initially, a diagnostic laparoscopy was done and hernia repair was approached transabdominal. A supraumbilical port incision was made and a pneumoperitoneum was created (Figure 2).

**Figure 3:**
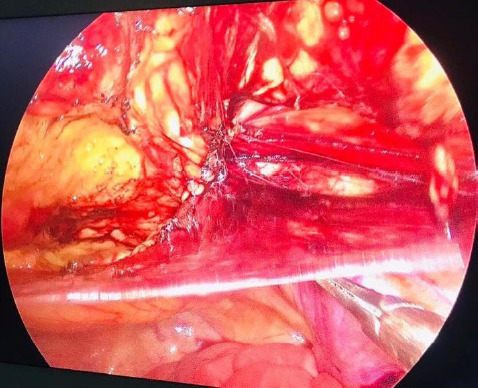
Laparoscopic image showing the creation of peritoneal flap.

There was a defect of 2.0 × 2.5 cm with reduced omentum as its content. The peritoneal flap was raised with a 4 cm margin around the hernia orifice. Prolene mesh was placed, the peritoneal flap was reduced and both were fixed with nonabsorbable tacks respectively (Figure 3).

The pneumoperitoneum was deflated and ports were closed after confirming hemostasis. The postoperative period was uneventful and she was discharged on the first postoperative day.

## DISCUSSION

Spigelian hernia is a rare ventral hernia that arises through the defect in the Spigelian fascia which is located between the muscle layers of the abdominal wall along the semilunar line also known as spontaneous lateral ventral hernia, interparietal, intermuscular, or intramural hernia.^1^ It constitutes approximately 1-2% of all hernias, with a higher incidence in females.^1^ It is reported that more than 90% of these hernias are located in the "Spigelian belt", which is a transverse 6-cm-wide zone in the lower abdominal wall because of the Spigelian aponeurosis which is the widest and weakest in this region.^2^ It arises when the intraabdominal pressure rises in conditions such as obesity, chronic cough, and peritoneal dialysis.^3^

Clinical presentation varies with the content of the hernia sac commonest being the pain. Also, a palpable mass in the anterior abdominal wall in case of larger hernias, and signs of incarceration with or without intestinal obstruction may present.^4^ Sometimes there is often no notable swelling as in our case making it difficult to diagnose clinically.^5^ The pain experienced by the patient was most probably due to a pull on the omentum. Imaging techniques like USG and CT scans have considerably aided in the diagnosis which is based on the demonstration of a hernial orifice in the Spigelian aponeurosis, an intramurally located hernial sac, andor sac content in the form of an intestine or omentum.^2^ Ultrasound is recommended as the first line imaging investigation, and CT scanning should be added in causes of doubt.^2-4^ In our case, USG did not reveal any specific findings so, a CT scan was performed that revealed a defect at Spigelian fascia with herniation of omental fat and small bowel loop.

Spigelian hernias are often confused with the lipoma or a parietal abscess.^5^ Only 50% of cases have been correctly diagnosed preoperatively.^6^ Major reasons for diagnostic difficulties are its low incidence with a maximum of 2% of abdominal wall hernias, a specific anatomical localization with intact external oblique aponeurosis covering the hernia sac, and a variable clinical presentation.^1,2^ Unusual presentations have also been reported involving the abdominal contents like the appendix, caecum, and terminal ileum.^7^ Some cases of congenital Spigelian hernia associated with undescended testis have also been reported.^8^

Due to the high rate of incarceration (up to 21%) and strangulation, the diagnosis of a Spigelian hernia is an indication of surgical repair, even in asymptomatic cases.^2^ The operation is usually simple to perform providing good results and low recurrence rate.^1^ The surgical approaches are open and laparoscopic.^9^ Laparoscopic repair is done for small defects while open repairs are done for larger defects. According to the recent European (EHS) and American (AHS) Hernia Societies guidelines, there are no definitive preferences between open and minimally invasive approaches and the decision is of the operating surgeon.^9,10^ Laparoscopic repair comprises placement of intraperitoneal onlay mesh (IPOM) or extraperitoneal mesh using transabdominal preperitoneal repair (TAPP) or total extraperitoneal repair (TEP) with use of mesh as mandatory.^9,10^ This hernia is ideally best suited to preperitoneal laparoscopic repair as the defect in the Spigelian aponeurosis is more clearly identified in the preperitoneal plane.^5^

Spigelian hernias are a rare, interparietal type of hernias. It remains a diagnostic challenge, due to the specific anatomic localization under the external oblique aponeurosis. Spigelian hernias are significantly at higher risk of incarceration compared to other types of abdominal wall hernias. Thus, even in asymptomatic cases, the management is surgical. Regardless of the surgical technique employed, a mesh repair is recommended.
